# Innovative Head-Mounted System Based on Inertial Sensors and Magnetometer for Detecting Falling Movements

**DOI:** 10.3390/s20205774

**Published:** 2020-10-12

**Authors:** Chih-Lung Lin, Wen-Ching Chiu, Ting-Ching Chu, Yuan-Hao Ho, Fu-Hsing Chen, Chih-Cheng Hsu, Ping-Hsiao Hsieh, Chien-Hsu Chen, Chou-Ching K. Lin, Pi-Shan Sung, Peng-Ting Chen

**Affiliations:** 1 Department of Electrical Engineering, National Cheng Kung University, Tainan City 701, Taiwan; n28041072@mail.ncku.edu.tw (W.-C.C.); n28031124@mail.ncku.edu.tw (T.-C.C.); n26070295@mail.ncku.edu.tw (Y.-H.H.); n28041056@mail.ncku.edu.tw (F.-H.C.); n28051132@mail.ncku.edu.tw (C.-C.H.); 2 Garmin Asia Corporation, New Taipei City 221, Taiwan; Richard.hsieh@garmin.com; 3 Department of Industrial Design, National Cheng Kung University, Tainan City 701, Taiwan; chenhsu@mail.ncku.edu.tw; 4 Department of Neurology, National Cheng Kung University Hospital, Tainan City 704, Taiwan; cxl45@mail.ncku.edu.tw (C.-C.K.L.); pishansung@gmail.com (P.-S.S.); 5 Department of Biomedical Engineering, National Cheng Kung University, Tainan City 701, Taiwan; chen@ncku.edu.tw

**Keywords:** fall detection, orientation filter, triaxial accelerometer, triaxial gyroscope, triaxial magnetometer, signal detecting and processing, head-mounted devices

## Abstract

This work presents a fall detection system that is worn on the head, where the acceleration and posture are stable such that everyday movement can be identified without disturbing the wearer. Falling movements are recognized by comparing the acceleration and orientation of a wearer’s head using prespecified thresholds. The proposed system consists of a triaxial accelerometer, gyroscope, and magnetometer; as such, a Madgwick’s filter is adopted to improve the accuracy of the estimation of orientation. Moreover, with its integrated Wi-Fi module, the proposed system can notify an emergency contact in a timely manner to provide help for the falling person. Based on experimental results concerning falling movements and activities of daily living, the proposed system achieved a sensitivity of 96.67% in fall detection, with a specificity of 98.27%, and, therefore, is suitable for detecting falling movements in daily life.

## 1. Introduction

Falling accidents that involve elderly people are an important issue in home care [1,2,3,4,5,6,7,8,9,10,11,12,13], because 28~35% of people over 65 years old have falling accidents more than once every year [6,8,10], and 10~20% of those suffer from injuries that involve hospitalization or death [5]. Without assistance, most elderly people cannot raise themselves and so remain on the ground after a fall, leading to serious physiological effects with enormous medical costs [3,7,14]. To monitor falling accidents, several systems that exploit a variety of methods, such as computer vision and motion sensing, have been proposed [1,2,3,4,5,6,7,8,9,10,11,12,13]. The most common means of fall detection involve placing spatially distributed sensors (including cameras [1,3,7], microphones [2,3], and floor sensors [11,12]) within the elderly person’s house to establish a smart home environment, in which a falling accident is identified by image, audio and vibration sensors. Yu et al. [1] proposed a fall detection system that is based on computer vision for home care of the elderly. This system can distinguish among human postures and the objects in the room to locate an elderly person who has fallen. However, this system functions correctly only when the person is in the area where the sensors are mounted with the absence of obstacles in the sensing range. Therefore, several systems have been developed using wearable devices; they have good portability without being limited to a particular region [4,5,6,9,10,13,15]. The accelerometer, gyroscope, and magnetometer are sensors that are commonly integrated into unobtrusive products as wearable devices due to their small size and low power consumption. These sensors contain Microelectromechanical Systems (MEMS), which apply different measuring structures for corresponding physical parameters such as acceleration, angular velocity, and magnetic field magnitude. [16,17,18]. Based on the measurements, the system can estimate the acceleration and orientation of the human body part for rehabilitation, posture recognition, and fall detection [4,5,6,9,10,13,16]. Palmerini et al. [4] developed a fall detection method that is based on wavelet analysis, which determines whether the data that are captured by the accelerometer correspond to a fall by recognizing features with reference to a model of falling movements. However, this method requires information about numerous characteristics of falling movements and manual adjustments to wavelet parameters to minimize the rate of false alarms in fall detection. Therefore, variation among people and within the environment make this method difficult to implement for real-life fall detection. Sabatini et al. [5] proposed a fall detection system that transmits data from an accelerometer, a gyroscope, and a barometric altimeter to a smartphone via Bluetooth and then uploads those data from the smartphone to a notebook via Wi-Fi. From the uploaded data, the vertical velocity and the change in height are estimated using an extended Kalman filter and a complementary filter, to identify falling accidents by comparison with relevant thresholds. However, the Kalman filter has high computational requirements and requires a high sampling rate for the sensor. Moreover, a huge number of data are transmitted, causing the system to have high power consumption, making it unsuitable for long-term monitoring where computation and communication resources are limited.

This work proposes a head-mounted system for detecting falling movements of elderly people. For the comfort of the elderly person, the appearance of proposed system is similar to common eyeglasses and suitable for long-term monitoring. Since the system is worn on the head, motion does not vary dramatically during everyday activities; falling movements are recognized by monitoring the posture of the human head and comparing Root Mean Square (RMS) acceleration and changes in pitch and roll angles, with threshold values. To eliminate errors and calibrate the estimation of angles, a Madgwick’s filter is used to correct the drift of the gyroscope using data from the accelerometer and the magnetometer. Experimental results verify that the proposed system has high accuracy in detecting falls by elderly people and can immediately transmit alarm messages through a wireless network to the data server when a falling accident is detected.

With respect to the remaining sections of this paper, Section 2 presents the hardware that is adopted in our system, methods for detecting falls, and the experimental setup for evaluating the proposed system. Section 3 presents the RMS acceleration, and the filtered pitch and roll angles calculated from the data of the implemented sensors. Experimental results concerning falling and daily life activities are provided to verify the functionality of the proposed system. Finally, Section 4 presents conclusions and summarizes the purpose of our work.

## 2. Materials and Methods

### 2.1. Structure of Proposed System

**Figure 1:**
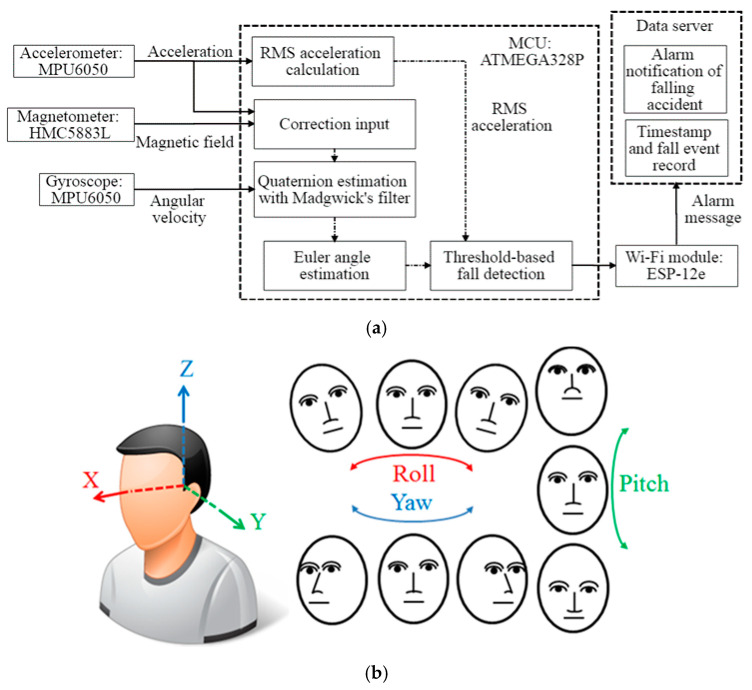
(**a**) Block diagram of proposed system; (**b**) Sensing axes of accelerometer, gyroscope, and magnetometer.

Figure 1 shows the structure of the proposed system consisting of an 8-bit Micro-Controller Unit (MCU), a 16-bit triaxial accelerometer, a 16-bit triaxial gyroscope, a 12-bit triaxial magnetometer, and a Wi-Fi module. The manufacturer, specifications, and configuration information of these components are shown in Table 1. The MCU (ATMEGA328P, Atmel, San Jose, CA, USA), with a 32 kB programmable memory, is driven by an internal resistance–capacitance oscillator at 8 MHz. The accelerometer and gyroscope are integrated into one chip (MPU6050, InvenSense, San Jose, CA, USA). Since the acceleration and angular velocity change dramatically during the fall, ±16 g and ±2000 °/s are selected in this work for the optimal resolution of the sensing results, where “g” is defined as the measurement of acceleration in terms of acceleration due to gravity. Additionally, a magnetometer (HMC5883L, Honeywell Aerospace, Phoenix, AZ, USA) is implemented in our system to measure the magnetic field to calculate the heading of the Earth’s magnetic field, pointing northward as the reference direction for angular calibration. The magnitude of the Earth’s magnetic field varies from 0.25 to 0.65 G in different locations [14]. A sensor-field range of ±0.9 G is chosen for our application, where “G” is the unit of measurement of magnetic induction. Since the proposed system uses the Earth’s magnetic field to provide the reference direction, variation therein does not affect the ability of the proposed system to detect a falling accident. However, when the sensor is saturated due to its close proximity to the ferromagnetic object, it exhibits a “hard iron error”, and the proposed system must conduct a sensor calibration. During the calibration, the magnetometer must be spun vertically and horizontally to find the maximum and minimum values of the magnetic field around the *X*-axis, *Y*-axis, and *Z*-axis. The environmental offsets along the three axes can be calculated from the averages of the maximum and minimum values of the magnetic field. Therefore, the hard iron error that is caused by nearby ferromagnetic objects can be removed by applying these environmental offsets to provide accurate measurements of the magnetic field. The sensed values of acceleration, angular velocity, and magnetic field magnitude are stored in the registers of MPU6050 and HMC5883L. The MCU will request these values via Inter-Integrated Circuit (I2C) communication to estimate the user’s posture every 50 milliseconds. Based on these sensed values, the RMS acceleration and orientation can be calculated to estimate the user’s posture. The system will analyze whether the user has fallen based on the proposed fall detection scheme and the sequence of the user’s posture. When a fall is detected, the Wi-Fi module (ESP8266, Expressif, Shanghai, China) is enabled and automatically connects to the Internet. Subsequently, an alarm message will be sent to be recorded as a fall event and initiate a notification of the falling accident to the emergency contact. The proposed system is designed for use indoors with Wi-Fi, including in the home, care centers, and hospitals. However, when the user is outdoors, the Wi-Fi signal can still be provided by the hotspot of a smartphone or public Wi-Fi.

Our system uses a 400 mAh Li-ion battery with a size of 20 mm × 30 mm × 7 mm. The voltage of the battery ranges from 2.7 to 4.2 V; as such, the battery is regulated to 3.3 V and powers the other components. A battery charging circuit that is based on TP4056 is implemented in the proposed system, so the device can be charged with 5 V from the common Universal Serial Bus (USB) power source.

The power consumption of the main components is presented in Table 2. To extend the battery life of the proposed system, the MCU is set to sleep mode to save power and is awoken into active mode by the built-in watchdog timer for periods of 30 ms. In active mode, the MCU takes about 20 ms to request measurements from the sensors and to calculate the RMS acceleration and orientation to estimate the posture of the user. The accelerometer is set to low-power mode, limiting the sampling rate to 20 Hz and reducing the current to 60 µA. The gyroscope and the magnetometer are operated in normal operation mode, and the currents through them are 3.60 mA and 100 µA, respectively. Despite the low sampling rate, a dramatic change in acceleration is still recognized to detect free-falling and an impact following a fall. Additionally, Madgwick’s filter is implemented in the proposed system to provide estimations of pitch, roll, and yaw angles, with a static error < 2 degrees and a dynamic error < 7 degrees when the sampling rate of the data is 10 Hz [19]. Therefore, fall detection can be achieved even at the low sensor sampling rate of 20 Hz. Moreover, since the Wi-Fi module consumes much power (120 mA in transmission), it is disabled by default (0.5 µA). Every 10 min, the Wi-Fi module will be enabled to send an “active status” message along with the battery level of the proposed system. The system also enables the Wi-Fi module and connects to a network to send a message concerning a falling accident whenever a fall has been detected. From the power consumption and operating cycle of the main components, the average current (~5.22 mA) can be obtained. Therefore, with a fully charged 400 mAh battery, the proposed system can monitor the user’s actions for at least 70 h. This duration indicates that the user does not need to frequently charge the proposed system and fall detection can continue reliably.

### 2.2. Fall Detection Scheme

This firmware of the proposed system was developed with Arduino Software (IDE) version 1.8.6 to provide an alarm notification when the elderly person has fallen. According to data from the accelerometer, gyroscope, and magnetometer, the fall detection scheme is achieved by analyzing the acceleration and orientation associated with falls. The selected fall detection techniques are unsophisticated and based on a comparison of acceleration and orientation with corresponding threshold values that are set in advance, owing to the limited storage and computing capacities of the MCU. Falling movements are identified only when the acceleration and the orientation of the upper body exceed preset thresholds that are set based on expert opinion or experiments. Figure 1b presents the posture of a human head relative to three axes (*X*-axis, *Y*-axis, and *Z*-axis); the spatial orientation is represented by pitch, roll, and yaw angles. As shown in Equation (1), the measured values of acceleration relative to the three axes are used to calculate the RMS acceleration, which represents the intensity of the triaxial acceleration of the user [8,20,21].

(1) $$RMS=\sqrt{Acc_{X}^{2}+Acc_{Y}^{2}+Acc_{Z}^{2}}$$

The static RMS acceleration is approximately the acceleration due to gravity (1 g) and varies with the movement of the sensor. Additionally, the human head’s spatial orientation is considered as the feature of the falling movement. When the elderly person loses balance and experiences a fall, an angular variation of the human body begins and the orientation will stop until the elderly person hits the ground or low-level objects. In the ideal case, integrating the angular velocity from a three-axis gyroscope can provide an estimate of the correct orientation of the human head. In fact, the error in the angular velocity that is acquired from gyroscope measurements accumulates, leading to a drift of the estimated angles [22]. Therefore, an orientation filter is utilized to accurately estimate angles based on the data from the accelerometer, the gyroscope, and the magnetometer [19,23]. Generally, the Kalman filter is a common solution for an orientation filter, which yields a better angular prediction based on current observations and previous angular estimates. However, Kalman filters need a high rate of computation and sensor responses. Another solution is the complementary filter, which represents an uncomplicated solution for angular estimation. It uses low-pass and high-pass filters to remove high-frequency noise (accelerometer and magnetometer) and low-frequency noise (gyroscope) [24,25,26]. The filtered signals are combined to yield an estimate of the orientation. However, the characteristics of the disturbances may vary among scenarios, yielding inaccurate angle estimation [25]. Therefore, in this work, an orientation filter proposed by Madgwick et al. was used. This filter has a lower computational load than the Kalman filter and can be operated at a low clock rate (8 MHz) and with a low-power-consumption (average, 12 mW) MCU with less computational time to avoid any delay in the processing of the sensor data [27]. Additionally, compared with the complementary filter, Madgwick’s filter minimizes the disturbances from the accelerometer and gyroscope by the gradient descent method. Madgwick’s filter minimizes these disturbances using the gradient descent method. The local magnetic field that affects the estimate of orientation is removed by reducing the constraint on the rotation of the magnetic field vector [19,26]. Therefore, Madgwick’s filter yields more accurate estimates of orientation than does the complementary filter without excessively increasing the computational requirements.

The quaternion is applied to provide information about the orientation of the accelerometer, gyroscope, and magnetometer to Madgwick’s filter [19]. This method has several advantages; for example, no Gimbal Lock issue arises and the computational requirements are lower than those for computing Euler angles. A quaternion (*q*) is composed of a real part and a three-dimensional imaginary part, and can be viewed as an extension of a complex number to four-dimensional space [28,29,30], consistent with Equation (2).

(2) $$q=q_{0}+q_{1}i+q_{2}j+q_{3}k$$

where $q_{0}$, $q_{1}$, $q_{2}$, and $q_{3}$ are real numbers; i, j, and k are the imaginary units along the three spatial axes and satisfy the following rules: $i^{2}=j^{2}=k^{2}=-1$ and ijk=−1.

As shown in Equations (3) and (4), Madgwick’s filter estimated the current quaternion orientation ($q_{est,t}$) from its value in the previous iteration ($q_{est,t-1}$) and the rate of orientation change (${\dot{q}}_{est,t}$), which is obtained from the data from the accelerometer, the gyroscope, and the magnetometer [18,30].

(3) $$q_{est,t}={\hat{q}}_{est,t-1}+{\dot{q}}_{est,t}\cdot \Delta t$$

(4) $${\dot{q}}_{est,t}={\dot{q}}_{gyro,t-1}-\beta \left({\dot{q}}_{acc,t-1}+{\dot{q}}_{mag,t-1}\right)$$

where Δt is the sampling period. β is a well-chosen constant between 0 to 1 that is used to adjust the rate of the correction that is provided by data from the accelerometer and the magnetometer. The ${\dot{q}}_{gyro,t-1}$, ${\dot{q}}_{acc,t-1}$, and ${\dot{q}}_{mag,t-1}$ derive their quaternion data from the gyroscope, accelerometer, and magnetometer in the preceding iteration, respectively. The definition of ${\dot{q}}_{gyro,t-1}$ eliminates the gyroscope bias error, and ${\dot{q}}_{acc,t-1}$ and ${\dot{q}}_{mag,t-1}$ can be obtained in the Jacobian matrix to compensate for accumulated errors in the gyroscope; it can be expressed as in Equations (5)–(7) [30].

(5) $${\dot{q}}_{gyro,t}=\frac{1}{2}q_{est,t-1}⊗\left(\omega_{r,t}-B_{g}\right)$$

(6) $${\dot{q}}_{acc,t}=J_{a}a_{u,t}{|}_{J_{a}=\frac{dg_{a,t}}{dq}}$$

(7) $${\dot{q}}_{mag,t}=J_{m}m_{u,t}{|}_{J_{m}=\frac{ds_{i,t}}{dq}}$$

where $\omega_{r,t}$ and $B_{g}$ are the raw gyroscope data at the current iteration and the gyroscope bias error, $a_{u,t}$ is used to remove gravity acceleration and bias errors from the accelerometer data, and $m_{u,t}$ is used to remove the soft iron effect and hard iron effect. Therefore, as shown in Equation (4), given the derived quaternion data from the gyroscope and the compensation of the orientation from the derived quaternions of the accelerometer and magnetometer, the rate of orientation change (${\dot{q}}_{est,t}$) is accurately obtained to update the estimation of the quaternion, and the correct estimated quaternion ($q_{est}$) is also integrated. The roll, pitch, and yaw angles are calculated from the scalar and other components of the estimated quaternion ($q_{est}$) according to the following equations [14,18].

(8) $$Roll=a\tan2(\frac{2\times q_{1}\times q_{2}-2\times q_{0}\times q_{3}}{2\times q_{0}{}^{2}+2\times q_{1}{}^{2}-1})$$

(9) $$Pitch=-\sin^{-1}(2\times q_{1}\times q_{3}+2\times q_{0}\times q_{2})$$

(10) $$Yaw=a\tan2(\frac{2\times q_{2}\times q_{3}-2\times q_{0}\times q_{1}}{2\times q_{0}{}^{2}+2\times q_{3}{}^{2}-1})$$

**Figure 2:**
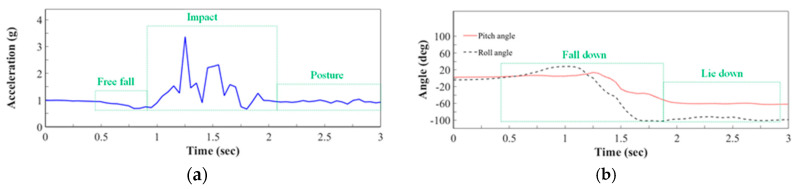
(**a**) Root Mean Square (RMS) acceleration during falling movement; (**b**) Pitch and roll. angles during falling movement.

Figure 2 plots the waveforms of simulated falls that are recorded by the system when a mannequin is used to mimic an elderly person to reveal the process during the falling movement. The three periods of a fall are defined as the “Free Fall”, “Impact”, and “Posture” periods. When the fall starts, the elderly person is free-falling, corresponding to a low RMS acceleration—that is, the “Free Fall” period. Then, in the “Impact” period, the elderly person collides with the ground, yielding a high RMS acceleration. Moreover, after this impact, the bounce of the elderly person’s body yields several peaks. Finally, in the “Posture” periods, the RMS acceleration returns to gravity acceleration and the elderly person remains on the ground or furniture until someone provides assistance. The proposed system recognizes the “Free Fall” and “Impact” phases during the falling accident to determine whether a fall has occurred, by comparing the acceleration and orientation of the moving head with thresholds.

To determine the optimal thresholds for detecting the impact and posture during a fall, a preliminary experiment was conducted, and the data of 120 falling movements and 450 daily life activities were collected from five subjects with ages ranging from 21 to 28 years, weights ranging from 68 to 110 kg, and heights ranging from 170 to 178 cm. Based on the observations from these recorded data, at the start of the fall, the subject is free-falling, corresponding to a low RMS acceleration. The subject’s body then collides with the ground, yielding a high RMS acceleration. Therefore, the thresholds of the RMS acceleration are set to 0.9 g as the low value and 1.9 g as the high value, as this acceleration range can obviously distinguish falling movements. However, only 59.05% of movements can be identified correctly when RMS acceleration is considered. Therefore, the angular change and the period of the falling movement are taken into consideration for posture recognition during the falling accident to increase the accuracy of fall detection. According to observation of the collected acceleration data, low acceleration and high acceleration appeared in the first 2 s at the start of a falling movement. Additionally, in this period of 2 s, the average angular changes were around 40 to 125 deg/sec. To obtain the optimal threshold values in our method, Youden’s index was applied [31,32]. Different threshold combinations for the period (0.1~2 s) and angular change (40~125 deg/sec) were tested. A higher value of Youden’s index means that more falling movements can be recognized and there will be fewer false alarms with the tested threshold values. Therefore, through achieving the maximum value of the index, the optimal threshold values for the angular change in pitch and roll angles were identified and then set to 48 deg/sec and 0.7 s in our work.

**Figure 3:**
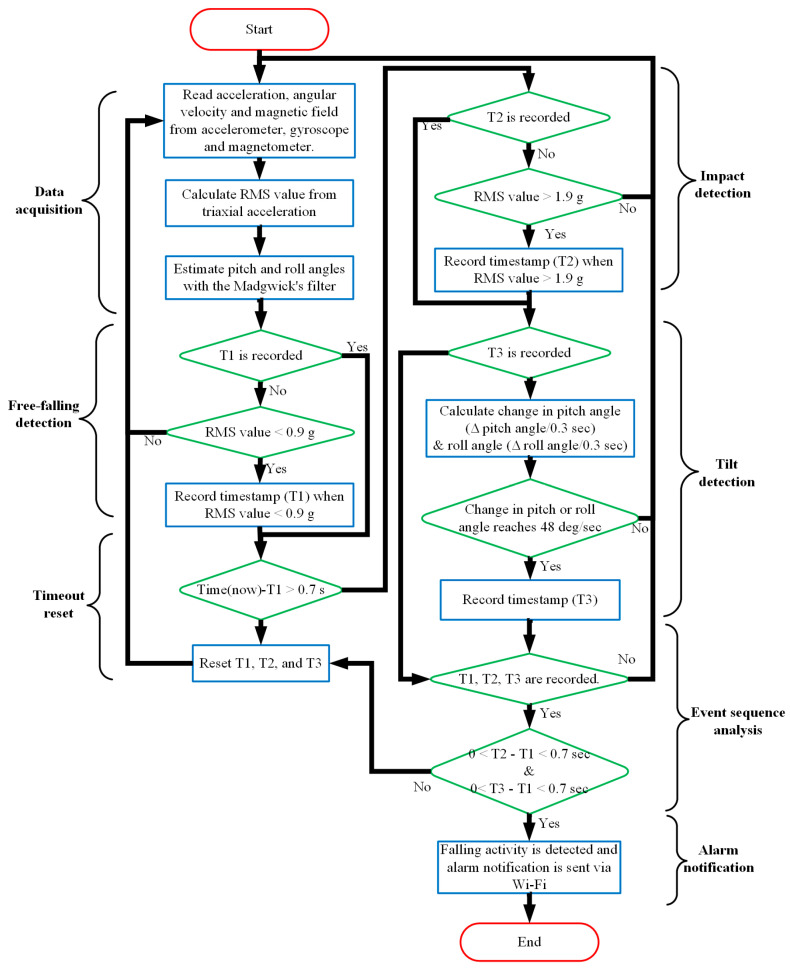
Flowchart of proposed scheme for threshold-based fall detection.

Figure 3 presents a flowchart of the proposed fall detection divided into the data acquisition stage, free-fall detection stage, timeout reset stage, impact detection stage, tilt detection stage, and alarm notification stage. During the data acquisition stage, the RMS acceleration and orientation of the user’s posture are estimated from the obtained acceleration, angular velocity, and magnetic field. Then, in the free-fall detection stage, when the RMS acceleration decreases to its low threshold (0.9 g), the system determines that a free-fall has begun and the timestamp (T1) of this free-fall event is recorded. In the timeout reset stage, the proposed system monitors the duration from the timestamp T1 to then. If no fall accident is detected for 0.7 s, then the system resets all the timestamps to their initial values. Next, in the impact detection stage, when the RMS acceleration rises over 2.5 g, the system identifies an impact and records a timestamp (T2). Simultaneously, the changes in pitch and roll angles in a period of 0.3 s are calculated to identify the posture of the user. When one of these angles changes faster than 48 deg/sec, a timestamp (T3) of the tilt event is recorded. Finally, if the times from T1 to T2 and from T2 to T3 are within 0.7 s, then the system determines that an accidental fall has occurred, so the alarm notification stage starts. The system will try to connect to a Wi-Fi network to send a message concerning the falling accident.

**Figure 4:**
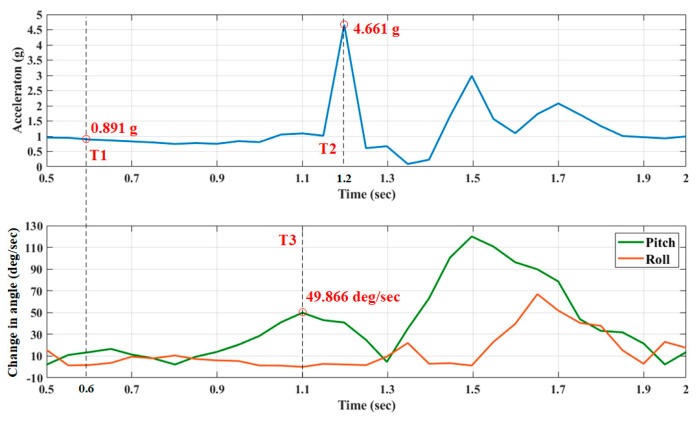
RMS acceleration and change in pitch and roll angles during a fall with marked points at which events are detected with associated timestamps.

Figure 4 plots the RMS acceleration and the angular change in pitch and roll during a fall with timestamps of detection. At timestamp T1 (0.6 s), the RMS acceleration is 0.891 g, fulfilling the condition for a free-fall event in the proposed fall detection scheme. Then, the RMS acceleration rises to 4.661 g, and an impact event at timestamp T2 (1.2 s) is recognized. Additionally, the angular change in the pitch angle reaches 49.866 deg/sec, and the tilt event is recorded as timestamp T3 (1.1 s). According to these thresholds for fall detection, the proposed system determines whether the acceleration and changes in angles are associated with a fall.

### 2.3. Falling and ADL Experimental Protocol

**Figure 5:**
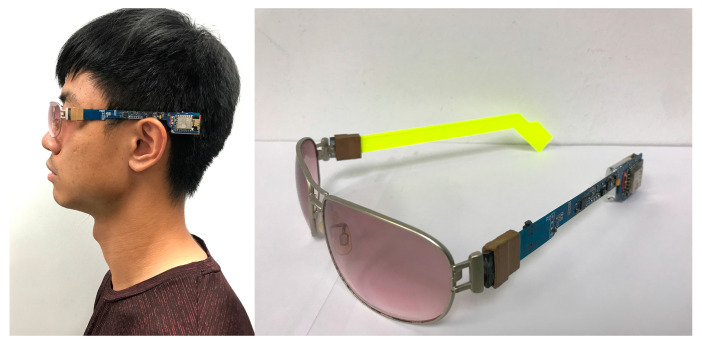
Photograph of prototype device.

Experiments were conducted on a prototype device as shown in Figure 5. A circuit board with dimensions of 137 mm × 22 mm was assembled on one side of the glass frame. The total mass of the proposed system with the battery was 37.4 g, which is close to the mass of a typical pair of regular eyeglasses, with lenses, weighing 17 to 50 g, where the “gram” is the unit of mass. A collaborative industrial design team designed a three-dimensional (3D) model of this frame. The team followed the design guidelines for head-worn and ear-worn products in anthropometry to improve the comfort of the proposed system.

Fifteen volunteers with ages ranging from 21 to 27 years, weights ranging from 50 to 110 kg, and heights ranging from 170 to 185 cm were invited to wear these eyeglasses for which the proposed system was implemented. Table 3 shows the falling movements and activities of daily living (ADLs) conducted by the test subjects. Each action or activity was carried out for five minutes and was taken from the everyday lives of elderly people, comprising 720 falling movements and 1500 ADLs. Since the direction of a fall is unpredictable, three common falls—trip falls, slip falls, and lateral falls—were selected. Two ADLs that involve large body movements, jumping and running, were used to determine whether the proposed system generated false alarms.

**Figure 6:**
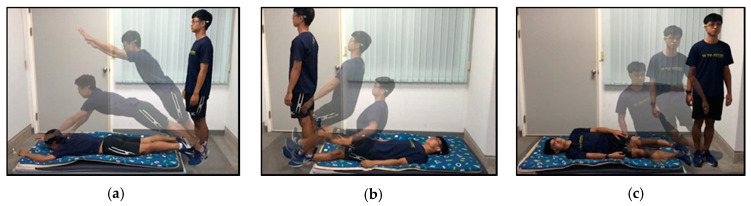
Deployment of test area and falling procedure in our falling experiment. (**a**) Trip fall. (**b**) Slip fall. (**c**) Lateral fall.

Figure 6 shows the deployment of the test area in our falling experiment, which involved a spongy mattress for the subject to fall on. The soft spongy mattress had a height of 10 cm and protected the subjects from injury. During the experiment, each subject was instructed to lose the balance of his/her body in the test area and fall in the indicated direction to simulate the falls.

### 2.4. Evaluation Metrics

To evaluate the performance of the proposed system, the six metrics of sensitivity, specificity, precision, accuracy, G-index, and F1-score were introduced. Sensitivity refers to the rate of correct detection of falling accidents. Specificity relates to the percentage of ADLs that are correctly recognized. Accuracy is defined as the rate of correct identifications of all falling movements and ADLs. The following equations provide the respective definitions.

(11) $$Sensitivity=\frac{TP}{TP+FN}$$

(12) $$Specificity=\frac{TN}{TN+FP}$$

(13) $$Accuracy=\frac{TP+TN}{TP+TN+FP+FN}$$

where a true positives (*TP*) are defined as the number of correctly detected falling movements, false positives (*FP*) are defined as the number of times that the system identifies an ADL as a falling movement, true negatives (*TN*) are defined as the number of times that an ADL is identified correctly, and false negatives (*FN*) are defined as the number of times the system does not detect a falling movement. Additionally, to analyze the classification performance of the proposed system, the other three metrics of precision, G-index, and F1-score were implemented in the evaluation. Precision represents the percentage of real falling accidents in relation to all detected falling accidents. Moreover, precision is often introduced with recall to emphasize the rate of true positives in the classification. Recall can be represented by the sensitivity as mentioned above, referring to the percentage correct detection of falling accidents. Moreover, to consider both precision and recall and provide a realistic measure of the test’s classification performance, the two average metrics G index and F1-score were applied. The G index is the geometric mean of the precision and recall. The F1-score is the harmonic mean of the precision and recall. The high values of these metrics mean good precision and good recall for the proposed method. The following equations provide the respective definitions.

(14) $$Precision=\frac{TP}{TP+FP}$$

(15) $$F_{1}-score=2\times \frac{Precision\times Recall}{Precision+Recall}=\frac{2\times TP}{2\times TP+FN+FP}$$

(16) $$G-index=\sqrt{Precision\times Recall}=\frac{TP}{\sqrt{\left(TP+FP\right)\times \left(TP+FN\right)}}$$

## 3. Results and Discussion

**Figure 7:**
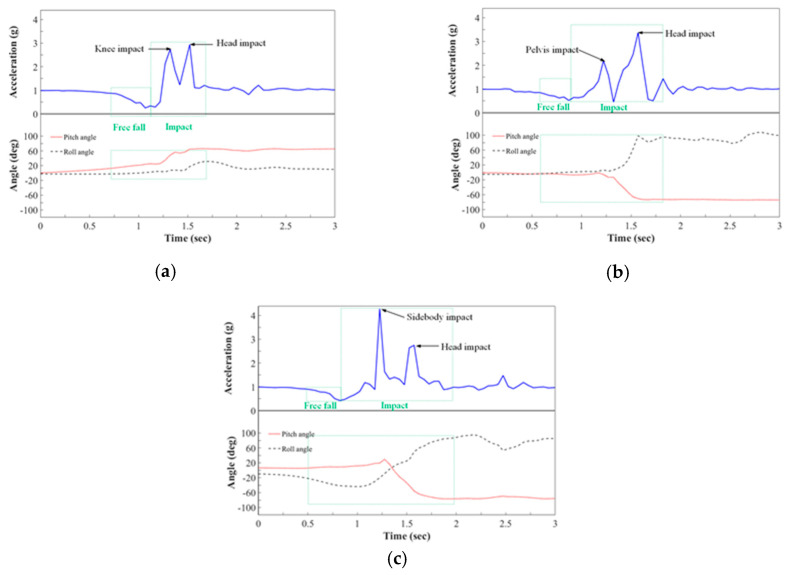
RMS acceleration along with pitch and roll angles during fall experiment. (**a**) Forward fall (trip); (**b**) Backward fall (slip); (**c**) Lateral fall.

In the experiments, the RMS acceleration, pitch angle, roll angle, and yaw angle of the subjects were recorded in a database to analyze fall detection performance. Figure 7 plots the measured waveforms of the three typical falls—(a) forward fall (trip), (b) backward fall (slip), and (c) lateral fall. The RMS acceleration is plotted as a solid blue line, and the values of the pitch and roll angles are plotted as a red dotted line and a gray dashed line. In the falling test, each subject’s knee, pelvis, or side collided with the ground, causing a small impact peak before the impact of the head; the subject then instinctively bent his or her body to maintain balance for protection, resulting in a brief rebound in the pitch angle. The peak value of RMS acceleration and the changes in the pitch and roll angles exceeded the threshold values, which were set to 0.9 g for the low value, 1.9 g for the high value, and 48°/s. Therefore, the characteristics in the free-fall and impact period can be clearly observed and the falling accidents can be detected correctly by the proposed system.

**Figure 8:**
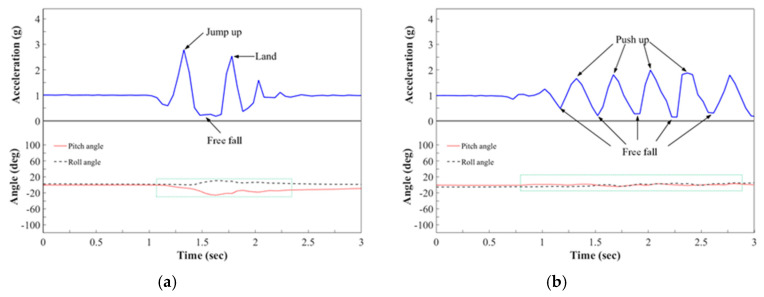
RMS acceleration and pitch and roll angles during ADL experiment. (**a**) Jump; (**b**) Stand–run–stand.

Figure 8 plots the RMS acceleration and the values of the pitch and roll angles of the ADLs. During jumping, two peaks in the waveform of the RMS acceleration were produced; one was caused by the acceleration associated with leaping, and the other was caused by the impact of landing. Since running is a repetitive motion, which can be separated into several small hops by the left and right feet, the waveforms of the RMS acceleration associated with running were similar to those for jumping, but the peaks were lower and more regular. When only the threshold value of RMS acceleration was used to detect a fall, these two activities, jumping and running, with large movements, were identified as falling movements. To reduce the misdetection of these activities, the changes in angles were considered. Although the RMS acceleration exceeded the threshold values of 0.9 g as the low value and 1.9 g as the high value, the changes in angles were not over the threshold value of 48°/s. Therefore, the fall could be distinguished from the activities of daily living. Based on experiments that involved three types of falling movement and two types of ADL, Table 4 shows the experimental results for the fifteen volunteers who performed the 720 falling movements and 1500 ADLs; 696 of the 720 falls and 1474 of the 1500 ADLs in the experiments were correctly identified. However, seven misdetections of falls might have resulted from the low-impact acceleration that was constrained by bodily behavior and the use of a soft mattress to protect the volunteers in the falling test from injury. Additionally, nine of the ADLs were mistaken as falls due to the forward leaning of the volunteer when he or she was landing as part of the running and jumping actions.

**Figure 9:**
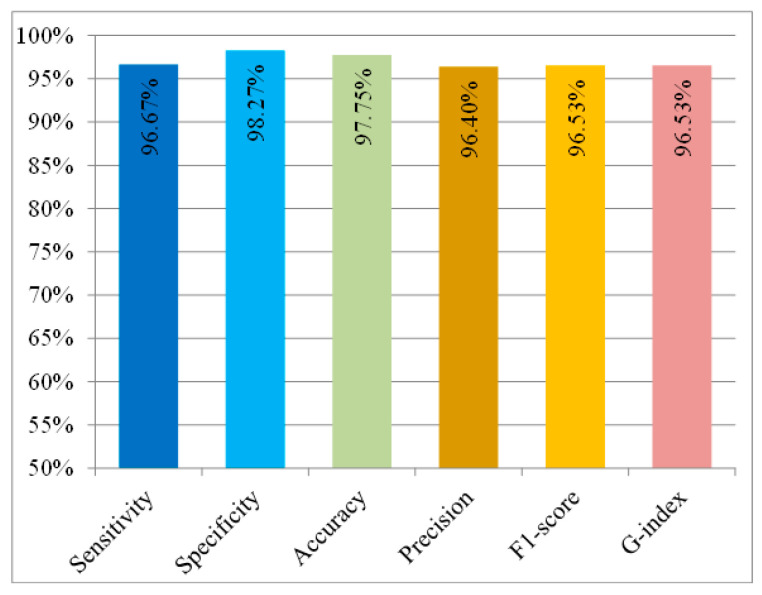
Metrics for evaluation of algorithm performance in falling tests and ADL tests (sensitivity, specificity, accuracy, precision, G index, and F1-score).

Based on the experimental results in Table 4, the sensitivity, specificity, precision, accuracy, G-index, and F1-score of the system were calculated as shown in Figure 9. The sensitivity and specificity of the system for all subjects exceeded 95%, indicating that the system can detect general falling movements with a false alarm rate of less than 5%. The proposed system was evaluated, with an average sensitivity of 96.67% in fall detection, a specificity of 98.27% in ADL detection, and an accuracy of 97.75% in identifying all activities, including falling movements and ADLs. Additionally, the misdetection rate for falls was lower than 3.33%, and the false alarm rate was lower than 1.73%. The metrics G index and F1-score both reached at least 96%, indicating that the method has effectiveness in both precision and recall. These results also reveal that the proposed system has the potential for fall detection and can be utilized to identify falling by elderly people in real life.

Table 5 compares the sensor types, classifier, method of real-time detection, and achieved performance for the fall detection method in this work with those in previous studies [4,5,33]. Palmerini et al. [4] applied the wavelet transformation to data that were captured by the accelerometer and extracted all the features to compare their similarity with data of the standard mother wavelet to recognize a fall. However, this method is based on a public dataset and not implemented for real-time fall detection. Since the acceleration is the only threshold, activities that involve large body movements such as jumping and running can be mistaken for a fall, leading to a high false-alarm rate. Therefore, the sensitivity and the specificity of the system reach only 90% and 89.7%, respectively. To increase the postural information that can be applied in fall detection, Sabatini et al. [5] proposed a fall detection system that uses an accelerometer, a gyroscope, and a barometric altimeter to identify falling accidents based on vertical velocity and change in height. The system can achieve 100% specificity. However, the sensitivity reaching only 80% makes this system particularly prone to identifying critical falls as normal movements. Hussain et al. [33] adopted machine-learning algorithms in their system, which identify the features associated with falling accidents based on the data of the accelerometer and the gyroscope. A high sensitivity of 99.44% was achieved, and their system had a specificity of 100%. However, this system requires a high sampling rate for the sensor data and has a high computational load, leading to high power consumption. Therefore, long-term fall monitoring is difficult in real life. In our work, a prototypic head-worn system, based on an accelerometer, gyroscope, and magnetometer, was developed to monitor the posture of the wearer. The sensors are operated at a low sample rate of 20 Hz to reduce the power consumption of our system. The data from the preliminary experiment were used to determine the optimal values of low acceleration, high acceleration, angular change, and event period to set as thresholds in this work. With these optimal thresholds, the proposed system achieves a sensitivity of 96.67% in fall detection, with a specificity of 98.27%, indicating that the proposed system is accurate and effective in fall detection, even with the limited sampling rate for the sensor data and low computational resources.

Nevertheless, despite the high-accuracy fall detection being achieved by the proposed system, this system is still an early-stage proof of concept for fall detection for the elderly. Further efforts are underway to refine the system and improve its accuracy for fall detection. To validate the system with more subject data and access to more realistic movements of elderly people, we have established approval for human subject research from the Institutional Review Board (IRB) of National Cheng-Kung University hospital. Additionally, the postural data collections of the elderly’s abnormal movements and daily movements in the hospital are ongoing. Based on these collected data, fall-risk movements of the elderly can be analyzed. The technique of the proposed system can be improved to accurately predict possible falling accidents and extended to several related applications for the well-being of the elderly, such as anti-fall notifications, rehabilitation advice, and analysis of long-term habits.

## 4. Conclusions

This work developed a head-mounted system with the appearance of eyeglasses for detecting falling accidents among the elderly; it was validated by an experiment involving fifteen volunteers. In this system, the sensors are integrated into eyeglasses that offer high portability and will not cause discomfort during usage for the elderly. The methods of the RMS and Madgwick’s filter are adopted to estimate the intensity of acceleration in three axes and accurate angles of the user’s head movement, respectively. Based on the RMS, the acceleration, the pitch angle, and the roll angle of head movement, the proposed system can recognize a falling accident by comparison with the prespecified thresholds and then notify the emergency contact via Wi-Fi. Based on experimental results with 720 falling movements and 1500 ADLs, most of the falling movements and ADLs could be correctly detected by the proposed system. The sensitivity reached 96.67% in detecting falls in different directions, and the specificity reached 98.27% in identifying ADLs. Rates of misdetection and false alarms under 3.33% and 1.73%, respectively, were also revealed. Therefore, the proposed system can achieve an accuracy of 97.75%, delivering an innovative and promising technique for detecting falls among elderly people in their daily lives.

## Figures and Tables

**Table 1:** Manufacturer, specifications, and configuration information of applied components.

	Component Name	Manufacturer	Specifications and Configuration
**MCU**	ATMEGA328P-MU	Atmel, San Jose, CA, USA	Internal resistance–capacitance oscillator at 8 MHz
**Accelerometer**	MPU6050	InvenSense, San Jose, CA, USA	Full-scale range: Accelerometer: ± 16 g Gyroscope: ± 2000°/s I2C speed: 400 kHz
**Gyroscope**			
**Magnetometer**	HMC5883L	Honeywell Aerospace, Phoenix, AZ, USA	Field range: ± 0.9 G I2C speed: 400 kHz
**Wi-Fi module**	ESP8266 (ESP-12e)	Expressif, Shanghai, China	Standard: IEEE 802.11b/g/n Bandrate: 115,200 bits/s
**Regulator**	MIC5219	Microchip, Chandler, AZ, USA	Regulated voltage: 3.3 V Maximal current: 500 mA
**Bi-ion battery**	702030	-	Capacity: 400 mAh Size: 20 mm × 30 mm × 7 mm
**Battery charger**	TP4056	TPower Semiconductor, Shenzhen, China	Charging current: 500 mA

**Table 2:** Current consumption and operation mode of main components implemented in proposed system.

Component	Mode	Current Consumption	Period
**MCU**	Active mode	3.58 mA (3.3 V @ 8 MHz)	20 ms out of every 50 ms
	Sleep mode with watchdog timer enabled	4.5 µA (3.3 V @ 8 MHz)	30 ms out of every 50 ms
**Accelerometer**	Low-power mode	60 µA (20 Hz sampling rate)	Always on
**Gyroscope**	Normal operation mode	3.60 mA	Always on
**Magnetometer**	Normal operation mode	100 µA	Always on
**Wi-Fi module**	Transmission mode (Tx 802.11n, −65 dBm)	120 mA	100 ms out of every 600 s
	Power down mode	0.5 µA	599.9 s out of every 600 s

**Table 3:** Falling movements and activities of daily living conducted in experiments.

Falling Movements	Forward Fall	At First Kneeling Down, Ending up Lying Down.
	Backward Fall	At First Impacting on Pelvis, Ending up Lying Down.
	Lateral Fall	Ending up Lying Down.
Activities of daily living (ADLs)	Running	
	Jumping	

**Table 4:** Experimental results of falling tests and ADL tests.

	Forward Fall	Backward Fall	Lateral Fall	Run	Jump
Subject 1	16/16	14/16	16/16	50/50	50/50
Subject 2	16/16	16/16	16/16	50/50	48/50
Subject 3	13/16	16/16	16/16	50/50	50/50
Subject 4	16/16	16/16	16/16	50/50	50/50
Subject 5	14/16	16/16	16/16	48/50	43/50
Subject 6	8/16	16/16	16/16	50/50	50/50
Subject 7	16/16	16/16	16/16	49/50	49/50
Subject 8	16/16	16/16	16/16	47/50	48/50
Subject 9	16/16	16/16	16/16	50/50	48/50
Subject 10	10/16	16/16	15/16	50/50	50/50
Subject 11	15/16	15/16	16/16	49/50	50/50
Subject 12	16/16	16/16	16/16	50/50	50/50
Subject 13	16/16	16/16	16/16	50/50	50/50
Subject 14	16/16	16/16	16/16	49/50	46/50
Subject 15	16/16	16/16	16/16	50/50	50/50
Total	220/240	237/240	239/240	742/750	732/750

**Table 5:** Comparison between proposed and previously developed fall detection systems.

Ref. (Year)	Sensor Type	Sensor Location	Classifier	Sample Rate	Real-Time Detection	Performance
**Palmerini, L.** [4] **(2015)**	Accelerometer	Lower back	Wavelet analysis and threshold-based algorithm	100 Hz	No	Sensitivity: 90% Specificity: 89.7%
**Sabatini, A.M.** [5] **(2016)**	Accelerometer Gyroscope Barometric	Right anterior iliac spine	Threshold-based algorithm	50 Hz	Yes	Sensitivity: 80% Specificity: 100%
**Hussain, F.** [33] **(2019)**	Accelerometer Gyroscope	Waist	Machine-learning-based algorithm	200 Hz	Yes	Sensitivity: 99.44% Specificity: 100%
**Our work**	Accelerometer Gyroscope Magnetometer	Head	Threshold-based algorithm	20 Hz	Yes	Sensitivity: 96.67% Specificity: 98.27%
